# Effect of cardiopulmonary bypass on activated partial thromboplastin time waveform analysis, serum procalcitonin and C-reactive protein concentrations

**DOI:** 10.1186/cc8166

**Published:** 2009-11-13

**Authors:** Bertrand Delannoy, Marie-Laurence Guye, Davy Hay Slaiman, Jean-Jacques Lehot, Maxime Cannesson

**Affiliations:** 1 Department of Anesthesiology and Intensive Care, Hospices Civils de Lyon, Louis Pradel Hospital, Claude Bernard Lyon 1 university, ERI 22, 28 avenue du doyen Lepine, 69500 Bron, France; 2 Department of Anesthesiology & Perioperative Care, School of Medicine, University of California, Irvine, 333 City Boulevard West Side, Orange, CA 92868-3301, USA

## Abstract

**Introduction:**

Systemic inflammatory response syndrome (SIRS) is a frequent condition after cardiopulmonary bypass (CPB) and makes conventional biological tests fail to detect postoperative sepsis. Biphasic waveform (BPW) analysis is a new biological test derived from activated partial thromboplastin time that has recently been proposed for sepsis diagnosis. The aim of this study was to investigate the accuracy of BPW to detect sepsis after cardiac surgery under CPB.

**Methods:**

We conducted a prospective study in American Society of Anesthesiologists' (ASA) physical status III and IV patients referred for cardiac surgery under CPB. Procalcitonin (PCT) and BPW were recorded before surgery and every day during the first week following surgery. Patients were then divided into three groups: patients presenting no SIRS, patients presenting with non-septic SIRS and patients presenting with sepsis.

**Results:**

Thirty two patients were included. SIRS occurred in 16 patients (50%) including 5 sepsis (16%) and 11 (34%) non-septic SIRS. PCT and BPW were significantly increased in SIRS patients compared to no SIRS patients (0.9 [0.5-2.2] vs. 8.1 [2.0-21.3] ng/l for PCT and 0.10 [0.09-0.14] vs. 0.29 [0.16-0.56] %T/s for BPW; P < 0.05 for both). We observed no difference in peak PCT value between the sepsis group and the non-septic SIRS group (8.4 [7.5-32.2] vs. 7.8 [1.9-17.5] ng/l; P = 0.67). On the other hand, we found that BPW was significantly higher in the sepsis group compared to the non-septic SIRS group (0.57 [0.54-0.78] vs. 0.19 [0.14-0.29] %T/s; P < 0.01). We found that a BPW threshold value of 0.465%T/s was able to discriminate between sepsis and non-septic SIRS groups with a sensitivity of 100% and a specificity of 93% (area under the curve: 0.948 +/- 0.039; P < 0.01). Applying the previously published threshold of 0.25%T/s, we found a sensitivity of 100% and a specificity of 72% to discriminate between these two groups. Neither C-reactive protein (CRP) nor PCT had significant predictive value (area under the curve for CRP was 0.659 +/- 0.142; P = 0.26 and area under the curve for PCT was 0.704 +/- 0.133; P = 0.15).

**Conclusions:**

BPW has potential clinical applications for sepsis diagnosis in the postoperative period following cardiac surgery under CPB.

## Introduction

Cardiac surgery using cardiopulmonary bypass (CPB) induces a non-specific acute inflammatory response. The pathophysiology of this inflammatory response is not completely understood [1,2]. Different mechanisms seem to be involved such as surgical trauma, transfusion, blood loss and hypothermia. CPB can activate the immune system via leucocyte interactions with the foreign surfaces of the CPB circuits [1]. Hemodynamic changes with ischemia-reperfusion and endotoxin release may also participate [1]. The term systemic inflammatory response syndrome (SIRS) has been proposed by the American College of Chest Physicians/Society of Critical Care Medicine Consensus Conference Committee to define a non-specific generalized inflammatory process independently from any causative factor [3].

Because of this non-specific SIRS situation, conventional clinical and biological tests fail to detect postoperative infection in the cardiac surgery setting. This can delay the diagnosis and treatment of sepsis and may increase postoperative mortality and morbidity [4]. Existing biological markers such as C-reactive protein (CRP) and procalcitonin (PCT) have been studied after CPB [5,6]. Serum CRP values increase during the postoperative period after cardiac surgery even in the absence of infection [7] and even if serum PCT seems to be a valuable marker of sepsis, its accuracy remains debatable [8,9] and its cost may be of concern.

In 1997, Downey and colleagues first described an abnormality in the optical transmission of the activated partial thromboplastin time (aPTT) [10,11]. This biphasic waveform (BPW) optical signal is related to the rapid formation of calcium-dependant complexes between very low-density lipoprotein and CRP [12]. Recently, several studies have suggested that BPW analysis is an easy, rapid and cost-effective tool for the diagnosis and prognosis assessment of severe sepsis patients in the intensive care unit (ICU) [13-15].

Kinetics and diagnostic value of BPW in the postoperative period following cardiac surgery under CPB have never been studied. The aim of this study was: to describe kinetics of BPW in the postoperative period following cardiac surgery under CPB; and to test its ability to discriminate patients with sepsis in the postoperative period following cardiac surgery under CPB.

## Materials and methods

### Study sample

We conducted a single-center prospective study between July 2007 and December 2007. The study protocol was approved by the institutional research ethics committee (Comité d'éthique des Hospices Civils de Lyon, Lyon, France). Written informed consent was obtained from each patient. The eligibility criteria were as follows: older than 18 years old, elective open-heart CPB surgery, American Society of Anesthesiology (ASA) physical status III or IV. Exclusion criteria were: preoperative SIRS of any cause (infection, systemic disease), corticoidsteroids or non-steroidal anti-inflammatory drug use within the last seven days before surgery.

### Data collection

Demographic data were recorded at inclusion: age, gender, weight, Simplified Acute Physiology Score II (SAPS II) [16], European System for Cardiac Operative Risk Evaluation (EuroSCORE) [17], and ASA physical status, left ventricular ejection fraction, and beta blocker prescription. Perioperative data were: type of surgery, aortic clamping time and CPB duration. Postoperative collected data were: ICU length of stay, need for inotropic or vasoactive support, postoperative complication as SIRS or sepsis, need for reoperation, hemorrhage (defined as blood loss of up to 4 ml/kg/hour in the postoperative setting), cardiac tamponade, acute kidney injury (increased serum creatininemia × 1.5 or urine output <0.6 ml/kg/h during six consecutive hours [18]), myocardial infarction (new Q waves of more than 0.04 seconds and 1 mm deep or a reduction in R waves of more than 25% in at least two continuous leads of the same territory). Clinical signs as heart rate, body temperature and respiratory rate were recorded every hour in the ICU and every eight hours in the step down unit. Biological data were recorded every day during the seven days following surgery.

### Perioperative management

General anaesthesia was induced using propofol and sufentanil. Muscular relaxant (cisatracurium) was administrated before tracheal intubation. Anaesthesia was maintained with inhaled sevoflurane or continuous propofol infusion depending on the anaesthesiologist choice. All patients had internal jugular central venous catheter for drug administration and central venous pressure monitoring. Electrocardiogram with ST monitoring, end-tidal carbon dioxide, arterial blood pressure using a radial artery catheter, and muscular relaxant monitoring were always used for monitoring. Pulmonary artery catheter was inserted when preoperative left ventricular ejection fraction was less than 0.4 or in the case of a preoperative severe pulmonary arterial hypertension (systolic pulmonary arterial pressure >50 mmHg). Antibiotic prophylaxis consisted of cefazolin 30 mg/kg at the induction and 1 g every four hours during surgery. Antifibrinolytic therapy (tranexamic acid 30 mg/kg) was administrated in every patient. Heparin (300 UI/kg) was administrated before CPB. Myocardial protection was performed with intermittent infusion of cold crystalloid cardioplegia. Tracheal extubation was performed in the surgical ICU when patients met all the required criteria according to the referring physician.

### Biologic measurements

Blood samples for biological measurements included white blood cell counts (WBC), PCT, CRP and BPW. Blood samples were drawn just after the induction and every morning during the seven days following surgery.

Serum CRP concentrations were measured using an immuno-turbidimetric assay on Modular analyser (Roche Diagnostics, Meylan, France). PCT was measured using the Kryptor PCT test (Brahms Diagnostica, Berlin, Germany). The aPTT wave form analysis was performed with the MDA II analyzer (BioMérieux, Marcy L'Etoile, France). In the aPTT assay, the slope of the initial phase of the light transmission profile quantifies an abnormal BPW. BPW signal unit is transmittance percentage per second (%T/s).

### Diagnosis of SIRS and sepsis

SIRS and sepsis diagnosis were established according to the American College of Chest Physicians/Society of Critical Care Medicine Consensus Conference Committee classification [3]. SIRS diagnosis requires the presence of two or more of the following criteria: body temperature above 38°C or below 36°C; heart rate above 90 beats/min; respiratory rate above 20 breaths/min or partial pressure of arterial carbon dioxide below 32 mmHg; leukocytes count above 12 G/L or below 4 G/L. Sepsis was defined as a SIRS associated to a documented infection. Pneumonia was defined as SIRS with infiltrate on chest radiograph and micro-organism isolated in bronchial secretions.

The final diagnosis of SIRS or sepsis was retrospectively established by two experts in taking into account the complete medical data. The experts were not in charge of the patients. The medical team in charge of the postoperative period was aware of the complete biological measurements except for BPW.

### Statistical analysis

All data were tested for normality with Shapiro-Wilk test. Data are reported as mean ± standard deviation or median (interquartile range) when appropriate. Data were analyzed using nonparametric Mann-Whitney U-test or Wilcoxon test as appropriate. The time course of CRP, PCT, and BPW concentration were tested by analysis of variance for repeated measures followed by a Bonferroni *post hoc *test. Patients were then divided in to three groups according to the predefined following criterion: patients presenting no postoperative SIRS (no SIRS group), patients presenting postoperative SIRS were defined as the SIRS group including patients presenting with sepsis, and patients presenting with non-septic SIRS. Receiver operating characteristic (ROC) curves were generated to test the ability of CRP, PCT, and BPW to discriminate between sepsis and non-septic SIRS patients varying the discriminating threshold of each parameters and area under the ROC curves were calculated and compared (MedCalc 8.0.2.0, MedCalc Software, Mariakerke, Belgium). A *P *value < 0.05 was considered statistically significant. All statistical analyses were performed using SPSS 13.0 for Windows (SPSS, Chicago, IL, USA).

## Results

**Figure 1:**
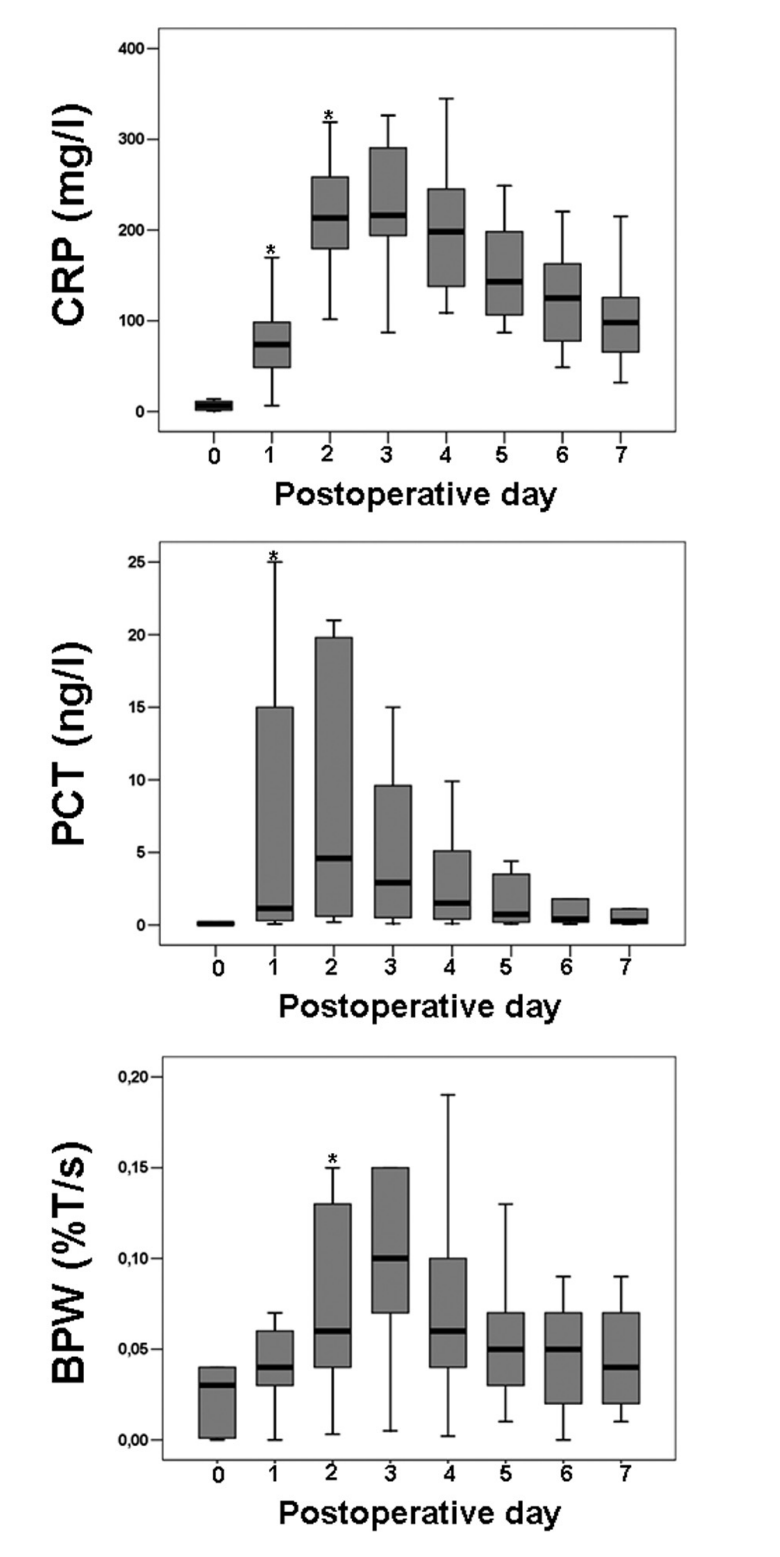
Box plot showing the evolution of CRP, PCT, and BWP in the studied sample. BPW = biphasic waveform; CRP = C-reactive protein; PCT = procalcitonin. D0, D1, D2, D3, D4, D5, D6, and D7: Preoperative and postoperative day 1, 2, 3, 4, 5, 6, and 7, respectively. * *P *< 0.05 compared with previous day.

Thirty two patients were included in the study. Demographic data are reported in Table 1. Sixteen patients (50%) did not present SIRS according to the predefined criteria. Among the 16 SIRS patients (50%), 5 patients (16%) presented with postoperative sepsis and 11 patients (34%) developed non-septic SIRS. CRP, PCT, and BPW postoperative evolutions in the 32 patients are presented in Figure 1. Interestingly, CRP and PCT were significantly increased at day 1 compared with baseline.

**Table 1:** Demographic data

Data	Total
**n**	32

**Gender: male/female**	25/7

**Age (years)**	72 (62-78)

**Weight (kgs)**	76 ± 11

**Length of stay in the ICU (days)**	5 (2-7)

**SAPS II score**	29 (23-38)

**EuroScore**	9 ± 3

**LVEF <40% (n)**	12 (38%)

**ASA physical status (III/IV)**	22/10

***Surgical procedure (n)***	
**- Valvular surgery**	15
**- Combined surgery**	5
**- Ascending aorta surgery**	10
**- CABG**	2

**CPB length (min)**	113 (86-121)

**Aortic cross clamping length (min)**	82 ± 28

***Post operative complications (n)***	
**- Total of patients presenting at least one postoperative complication**	18 (56%)
**- Non-septic SIRS**	11 (34%)
**- Sepsis**	5 (16%)
**- Reoperation**	4 (13%)
**- Hemorrhage**	7 (22%)
**- Inotropic support**	2 (6%)
**- Myocardial infarction**	6 (19%)
**- AKI requiring RRT**	3 (9%)

**Death (n)**	4 (13%)

Data expressed as mean ± standard deviation, median (interquartile range), or number (percentage).

AKI = acute kidney injury; ASA = American Society of Anesthesiology; CABG = coronary artery bypass grafting; CBP = cardiopulmonary bypass; EuroSCORE = European system for cardiac operative risk evaluation; ICU = intensive care unit; LVEF = left ventricular ejection fraction; RRT = renal replacement therapy; SAPS II = simplified acute physiology score; SIRS = systemic inflammatory response syndrome.

### Comparison between SIRS and no SIRS patients

Table 2 describes no SIRS vs. SIRS groups' characteristics. Length of stay was significantly lower in no SIRS group compared with SIRS group. Table 3 represents peak values for CRP, PCT and BPW in SIRS and no SIRS patients. We observed no difference in peak CRP value between no SIRS and SIRS groups (199 (180 to 264) vs. 240 (237 to 283) mg/l; *P *= 0.09) both values being statistically higher than CRP at baseline (*P *< 0.001 for both). On the other hand, we found that PCT and BPW peak values were significantly increased in SIRS compared with no SIRS (0.9 (0.5 to 2.2) vs. 8.1 (2.0 to 21.3) ng/l for PCT and 0.10 (0.09 to 0.14) vs. 0.29 (0.16 to 0.56) %T/s for BPW; *P *< 0.05 for both). Both PCT and BPW peak values corresponded with the day of sepsis diagnosis.

**Table 2:** no SIRS vs SIRS patients characteristics

Data	No SIRS group (n = 16)	SIRS group (n = 16)	*P *value
**Male/female**	12/4	13/3	0.986
**Age (years)**	74 (69-77)	69 (60-79)	0.46
**ASA physical status (III/IV)**	14/2	8/8	0.057
**EuroSCORE**	9 ± 3	9 ± 4	0.963
**LVEF <40%**	7 (44%)	5 (31%)	0.693
**Perioperative beta blockers**	4 (25%)	3 (27%)	0.74
***Baseline biologic measurements***			
**- WBC (G/L)**	7.6 ± 2.5	7.6 ± 2.1	0.817
**- CRP (mg/L)**	1.8 (1.1-5.7)	6.7 (2.2-14.3)	0.016
**- PCT (ng/L)**	0.07 (0.06-0.1)	0.09 (0.09-0.20)	0.052
**- BPW (%T/s)**	0.02 ± 0.02	0.03 ± 0.02	0.423
**SAPS II score**	29 (23-30)	32 (23-43)	0.221
***Surgical procedure***			
**- Valvular surgery**	5 (30%)	10 (62%)	_
**- Combined surgery**	2 (13%)	3 (19%)	
**- Ascending aortia surgery**	7 (44%)	3 (19%)	
**- CABG**	2 (13%)	0	
**CPB duration (min)**	106 (81-116)	120 (92-146)	0.09
**Aortic cross clamping time (min)**	75 ± 25	88 ± 31	0.187
**Length of stay in the ICU (days)**	2 (2-4)	5 (4-15)	0.006
***Post operative complication***			
**- Reoperation**	0	4 (25%)	0.11
**- Hemorrhage**	2 (13%)	5 (31%)	0.42
**- Tamponade**	0	2 (13%)	0.44
**- Inotropic support**	1 (6%)	5 (31%)	0.17
**- Myocardial infarction**	0	1 (6%)	0.97
**- AKI requiring RRT**	0	3 (19%)	0.22
**Death (n)**	0	4 (25%)	0.11

Data are expressed as mean ± standard deviation, median (interquartile range), or number (percentage).

AKI = acute kidney injury; ASA = American Society of Anesthesiology; BPW = biphasic waveform; CABG = coronary artery bypass grafting; CPB = cardiopulmonary bypass; CRP = C-reactive protein; EuroSCORE = European system for cardiac operative risk evaluation; ICU = intensive care unit; LVEF = left ventricular ejection fraction; PCT = procalcitonin; RRT = renal replacement therapy; SAPS II = simplified acute physiology score; SIRS = systemic inflammatory response syndrome; WBC = white blood cell.

**Table 3:** Peak value for CRP, PCT, and BPW according to the group

	**No SIRS (n = 16)**	**SIRS (n = 16)**	***P *value**
	
**Peak CRP (mg/L)**	199 (180-264)	240 (237-283)	0.09
**Peak PCT (ng/L)**	0.9 (0.5-2.2)	8.1 (2.0-21.3)	0.04
**Peak BPW (%T/s)**	0.10 (0.09-0.14)	0.29 (0.16-0.56)	0.03
	
	**SIRS group (n = 16)**		
		
	**SIRS (n = 11)**	**Sepsis (n = 5)**	
		
**Peak CRP (mg/L)**	239 (237-270)	270 (223-279)	0.37
**Peak PCT (ng/L)**	7.8 (1.9-17.5)	8.4 (7.5-32.2)	0.67
**Peak BPW (%T/s)**	0.19 (0.14-0.29)	0.57 (0.54-0.78)	0.003

Data are presented as median (interquartile range).

BPW = biphasic waveform; CRP = C-reactive protein; PCT = procalcitonin; SIRS = systemic inflammatory response syndrome.

**Table 4:** Non septic SIRS vs. septic patients' characteristics

Data	Non septic SIRS (n = 11)	Sepsis (n = 5)	*P *value
**Male/female**	8/3	5/0	0.55
**Age (years)**	69 ± 10	61 ± 24	0.37
**ASA physical status (III/IV)**	7/4	2/3	0.72
**Length of stay in the ICU (days)**	5 (3-6)	14 (8-18)	0.26
**SAPS II**	34 (27-42)	23 (19-50)	0.76
**EuroSCORE**	9 ± 4	8 ± 1	0.67
**LVEF <50%**	3 (27%)	2 (40%)	0.95
***Baseline biologic measurements***			
**- WBC (G/L)**	7.6 ± 2.4	6.9 ± 1.0	0.59
**- CRP (mg/L)**	6.4 (2.4-11.1)	10.9 (1.7-18.3)	0.96
**- PCT (ng/L)**	0.09 (0.08-0.18)	0.09 (0.07-0.20)	1
**- BPW (%T/s)**	0.02 ± 0.02	0.04 ± 0.03	0.27
***Surgical procedure (n)***			
**- Valvular surgery**	7 (63%)	3 (60%)	
**- Combined surgery**	2 (18%)	1 (20%)	
**- Ascending aorta surgery**	2 (18%)	1 (20%)	
**- CABG**	0	0	
**CPB duration (min)**	118 (90-120)	139 (103-208)	0.31
**Aortic cross clamping time (min)**	84 ± 27	99 ± 39	0.39
***Post operative complications (n)***			
**- Reoperation**	1 (9%)	3 (60%)	0.12
**- Hemorrhage**	2 (18%)	3 (60%)	0.27
**- Tamponade**	0	2 (40%)	0.15
**- Inotropic support**	3 (27%)	2 (40%)	0.95
**- Myocardial infarction**	0	1 (20%)	0.68
**- AKI requiring RRT**	1 (9%)	2 (40%)	0.43
**Death (n)**	2 (18%)	2 (40%)	0.75

Data are expressed as mean ± standard deviation, median (interquartile range), or number (percentage).

AKI = acute kidney injury; ASA = American Society of Anesthesiology; BPW = biphasic waveform; CABG = coronary artery bypass grafting; CPB = cardiopulmonary bypass; CRP = C-reactive protein; EuroSCORE = European system for cardiac operative risk evaluation; ICU = intensive care unit; LVEF = left ventricular ejection fraction; PCT = procalcitonin; RRT = renal replacement therapy; SAPS II = simplified acute physiology score; SIRS = systemic inflammatory response syndrome.

### Comparison between sepsis patients and non-septic SIRS patients

Among the 16 patients presenting with SIRS, five (31%) patients were classified as sepsis patients. Aetiology for sepsis was pneumonia in all patients. Its diagnosis occurred three (2.25 to 3.75) days after surgery. Sepsis and non-septic SIRS patient's characteristics are reported in Table 4. We observed no difference in peak CRP value between sepsis and non-septic SIRS groups (270 (223 to 279) vs. 239 (237 to 270) mg/l; *P *= 0.37) and no difference in peak PCT value between sepsis and non-septic SIRS groups (8.4 (7.5 to 32.2) vs. 7.8 (1.9 to 17.5) ng/l; *P *= 0.67; Table 3). On the other hand, we found that BPW was significantly higher in sepsis compared with non-septic SIRS (0.57 (0.54 to 0.78) vs. 0.19 (0.14 to 0.29) %T/s; *P *< 0.01).

### Ability of BPW to discriminate between sepsis and non-septic SIRS in the postoperative period following cardiac surgery

We found that a BPW threshold value of 0.465%T/s was able to discriminate between sepsis and non-septic SIRS with a sensitivity of 100% and a specificity of 93% (area under the curve: 0.948 ± 0.039; *P *< 0.01) Applying the previously published threshold of 0.25%T/s, we found a sensitivity of 100% and a specificity of 72% to discriminate between these two groups. Neither CRP nor PCT had significant predictive (area under the curve for CRP was 0.659 ± 0.142; *P *= 0.26 and area under the curve for PCT was 0.704 ± 0.133; *P *= 0.15).

## Discussion

This is the first study to focus on the perioperative kinetics of BPW in patients undergoing cardiac surgery under CPB. Our results show that postoperative BPW values have the potential to discriminate between sepsis and non-septic SIRS in this setting. A BPW threshold value of 0.465%T/s was able to discriminate between sepsis and non-septic SIRS with a sensitivity of 100% and a specificity of 93% (area under the curve: 0.948 ± 0.039; *P *< 0.01)

CPB induces a non-specific acute inflammatory response. Because of this non-specific SIRS situation, conventional clinical and biological tests fail to accurately detect infection in the postoperative period following cardiac surgery, inducing a delay in the diagnosis and treatment of postoperative sepsis. This issue is critical because mortality in sepsis remains high and because it has been demonstrated that early therapeutic intervention can improve prognosis [4]. On the other hand, indiscriminate use of antibiotics in all SIRS patients would lead to the development of resistant strains and increase toxicity and costs. This explains why it is of major importance to explore tools that can accurately discriminate between SIRS and sepsis in this setting.

Existing biological markers such as CRP and PCT have been studied after CPB [5,6]. Serum CRP values are not specific and have been shown to increase in the postoperative period following cardiac surgery even in the absence of SIRS [7]. Our data are consistent with this finding because CRP values were significantly increased in the no SIRS group compared with baseline. Moreover, the increase in CRP was not different between no SIRS and SIRS patients in our study. Serum PCT seems to be a valuable marker of sepsis but its accuracy remains debated in the postoperative period following cardiac surgery under CPB [8,9] and its cost may be of concern. In our study, we found that PCT was significantly increased after CPB but we found that this increase was significantly higher in SIRS group than in no SIRS group. However, PCT failed to discriminate between sepsis and non-septic SIRS patients in the present study.

The BPW has been proposed as a new tool for infection detection. Chopin and colleagues have demonstrated that BPW was more sensitive and more specific than PCT for the diagnosis of sepsis in the ICU [13] and more recently, Zakariah and colleagues have suggested that a combined evaluation with PCT would increase BPW specificity in sepsis identification [15]. The BPW is caused by calcium-dependent formation between lipoproteins and CRP [12]. The very low-density lipoprotein components from patients with BPW increase prothrombinase activity [19]. The BPW lasts two to three days and precedes the diagnosis of overt disseminated intravascular coagulation by 2.8 days on average [10,11] and could be a maladaptive consequence of the host haemostatic/endothelial responses [20-22]. The presence of an abnormality in the waveform pattern is independent of the aPTT clotting time [10] and not influenced by anticoagulants or coagulation factor deficiencies. Downey and colleagues [10] and Toh and colleagues [12] have shown that the BPW is not a surrogate marker for CRP or very low-density lipoprotein and provides additional information.

The BPW analysis is easy, and represents no additional cost compared with usual hemostasis tests that are preformed daily in the postoperative period following cardiac surgery. For instance, at our institution, a routine charge for an aPTT test is $7, a CRP test costs $9, and a PCT test costs $30 (US dollars).

**Figure 2:**
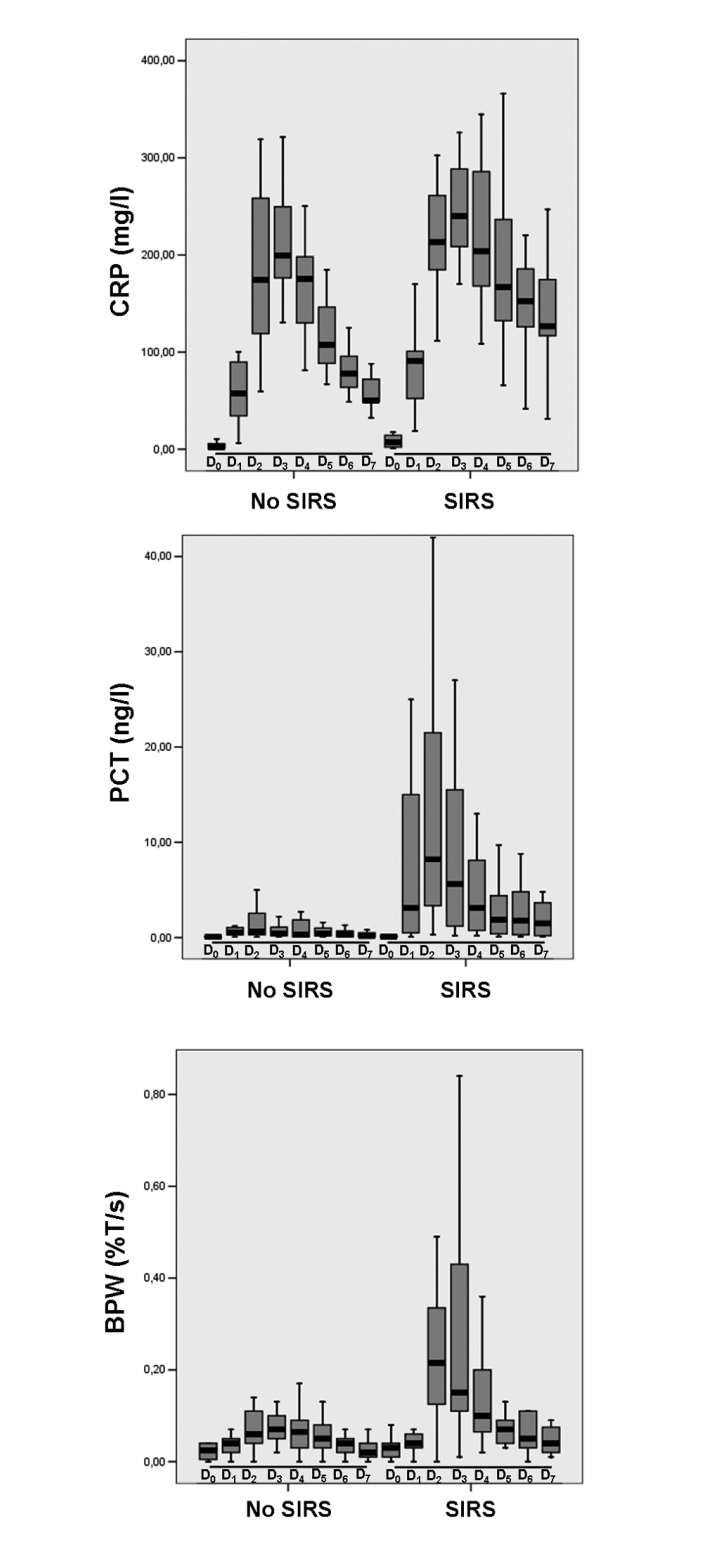
Box plot showing the evolution of CRP, PCT, and BWP in SIRS and No SIRS groups. BPW = biphasic waveform; CRP = C-reactive protein; PCT = procalcitonin; SIRS = systemic inflammatory response syndrome. D0, D1, D2, D3, D4, D5, D6, and D7: Preoperative and postoperative day 1, 2, 3, 4, 5, 6, and 7, respectively. * *P *< 0.05 compared with previous day.

**Figure 3:**
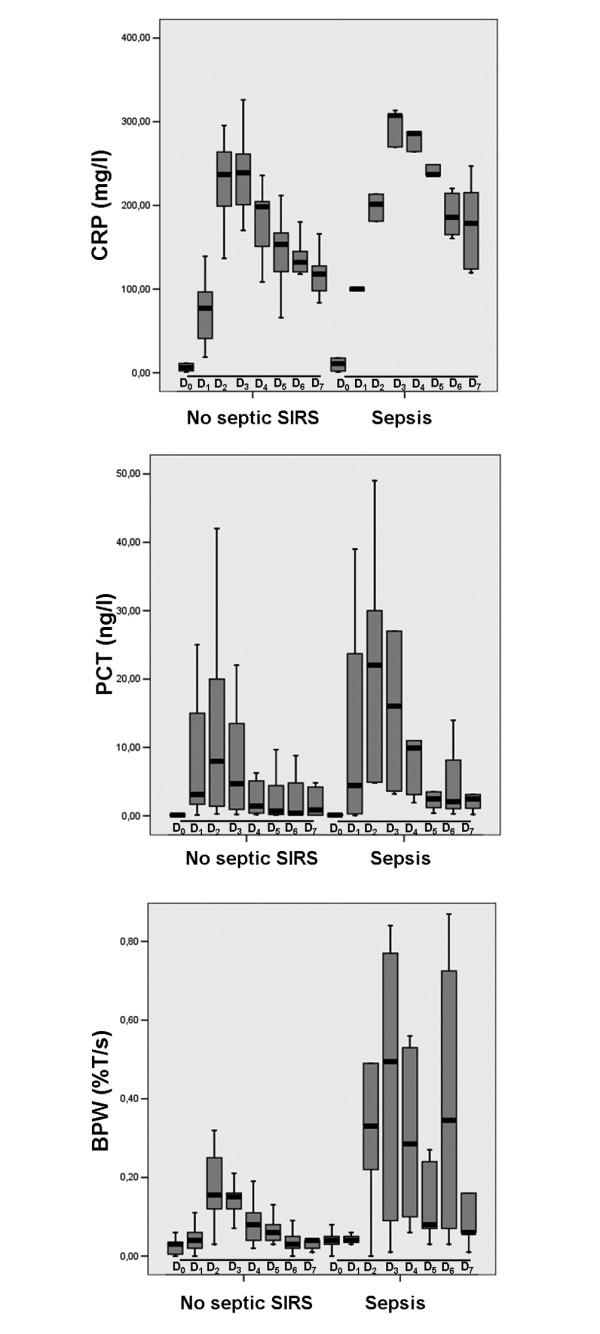
Box plot showing the evolution of CRP, PCT, and BWP in sepsis and Non septic SIRS groups. BPW = biphasic waveform; CRP = C-reactive protein; PCT = procalcitonin; SIRS = systemic inflammatory response syndrome. D0, D1, D2, D3, D4, D5, D6, and D7: Preoperative and postoperative day 1, 2, 3, 4, 5, 6, and 7, respectively. * *P *< 0.05 compared with previous day.

In the present study, we found that in the whole population the BPW was significantly increased during the first 48 hours following cardiac surgery compared with baseline (Figure 1). However, we observed no significant increase in the BPW in no SIRS patients whereas the increase in the BPW was significant in SIRS patients (Figure 2). This is similar to what was observed for PCT [23] and different from what was found for CRP that demonstrated a significant increase in both SIRS and no SIRS groups (Figure 2). More interestingly, when analysis was limited to SIRS group, we found that the increase in the BPW was significantly higher in sepsis patients compared with non-septic SIRS patients (Figure 3). At the same time, we observed no difference in PCT evolutions between sepsis patients and non-septic SIRS patients (Figure 3).

Whereas BPW was influenced by SIRS occurrence, its value remained below the existing infection thresholds in the no SIRS group. In the non-septic SIRS patients, the maximal value for BPW was 0.15 (0.12 to 0.25) %T/s on day 2 whereas infection threshold in ICU has been shown to be around 0.25%T/s [13]. On the other hand, every septic patient had BPW peak value above 0.25%T/s [13]. Consequently, we found that the best threshold value for BPW (i.e. threshold allowing for the best sensitivity and specificity) in the present setting was 0.465%T/s. This is probably related to the fact that BPW was significantly increased in non-septic SIRS patients. Consequently, the cut-off value for BPW in the postoperative period following cardiac surgery under CPB may be higher than the cut-off value used in conventional ICU.

### Study limitations

In the present study we did not include patients undergoing 'off-pump' cardiac surgery and no conclusion can be drawn regarding this population. However, cardiac surgery under CPB is the most challenging situation for postoperative sepsis diagnosis. Further studies will have to focus on off-pump cardiac surgery. An ideal marker of sepsis should be beneficial when employed in medical decision making. It remains to be determined what specific mechanism in sepsis produces an abnormal BPW and if the BPW has reliable clinical utility for determining risk, prognosis or treatment in the present setting.

We only studied 32 patients. This may not be enough to detect any statistically significant difference in peak CRP values as it is observed between the no SIRS and SIRS group. Further studies should include a larger number of patients to be conclusive.

Moreover, all of the sepsis patients in our study presented with pneumonia. Lungs are known to be a very good source of thromboplastin and this may explain part of our findings. Consequently, whether the BPW presents the same kinetics in other forms of lung injury (such as acid aspiration or smoke inhalation) or in other forms of sepsis (such as peritonitis) will need to be further explored.

Finally, a limitation of all studies on sepsis markers is that there is no gold standard to compare with. A list of potential signs and symptoms of sepsis was provided in the Sepsis Definitions Conference but none alone is specific for sepsis. However, this limitation applies to any other previously published studies on sepsis diagnosis.

## Conclusions

The diagnosis of early sepsis in CPB surgery postoperative condition is challenging. Usual biological test are often distorted by the occurrence of non-septic SIRS. In our experience, a BPW threshold value of 0.465%T/s was able to discriminate between sepsis and non-septic SIRS patients with a sensitivity of 100% and a specificity of 93% (area under the curve: 0.948 ± 0.039; *P *< 0.01). Consequently, the BPW seems to be an interesting marker for sepsis diagnosis in the postoperative period following cardiac surgery under CPB.

## Key messages

- Postoperative increase in BPW is significantly higher in patients with postoperative SIRS compared with patients with no postoperative SIRS.

- Postoperative increase in BPW is significantly higher in patients with postoperative sepsis compared with patients with non-septic postoperative SIRS.

- A BPW threshold value of 0.465%T/s was able to discriminate between sepsis and non-septic SIRS patients with a sensitivity of 100% and a specificity of 93% (area under the curve: 0.948 ± 0.039; *P *< 0.01).

- A routine charge for an aPTT test is $7, a CRP test costs $9, and a PCT test costs $30 at our institution.

- BPW seems to be an interesting marker for sepsis diagnosis in the postoperative period following cardiac surgery under CPB.

## Abbreviations

aPTT: activated partial thromboplastin time; BPW: biphasic waveform; CPB: cardiopulmonary bypass; CRP: C-reactive protein; EuroSCORE: European System for Cardiac Operative Risk Evaluation; ICU: intensive care unit; PCT: procalcitonin; ROC: receiver operator characteristics; SAPS II: Simplified Acute Physiology Score II; SIRS: systemic inflammatory response syndrome; WBC: white blood cell counts.

## Competing interests

The authors declare that they have no competing interests.

## Authors' contributions

BD was involved in the analysis and interpretation of data, drafting of the manuscript and final approval of the manuscript. M-LG was involved in the analysis and interpretation of data, drafting of the manuscript and final approval of the manuscript. DHS was involved in the analysis and interpretation of data, drafting of the manuscript and final approval of the manuscript. J-JL was involved in revising the manuscript critically for important intellectual content, editing the manuscript and final approval of the manuscript. MC was involved in conception and design, analysis and interpretation of data, editing the manuscript and final approval of the manuscript.
